# Sol-Gel-Prepared Nanoparticles of Mixed Praseodymium Cobaltites-Ferrites

**DOI:** 10.1186/s11671-016-1295-y

**Published:** 2016-02-09

**Authors:** Olga Pekinchak, Leonid Vasylechko, Iryna Lutsyuk, Yaroslav Vakhula, Yuri Prots, Wilder Carrillo-Cabrera

**Affiliations:** Semiconductor Electronics Department, Lviv Polytechnic National University, 12 Bandera Street, 79013 Lviv, Ukraine; Department of Chemical Technology of Silicates, Lviv Polytechnic National University, 12 Bandera Street, 79013 Lviv, Ukraine; Max-Planck-Institut für Chemische Physik fester Stoffe, Nöthnitzer Str. 40, 01187 Dresden, Germany

**Keywords:** Mixed cobaltites-ferrites, Perovskites, Nanopowders, Crystal structure, 61, 61.46.-w, 81.07.-b

## Abstract

Two series of nanocrystalline powders of PrCo_1 − *x*_Fe_*x*_O_3_ (*x* = 0.1, 0.3, 0.5, 0.7 and 0.9) of high purity were obtained by sol-gel citrate method at 700 and 800 °C. The formation of continuous solid solution with an orthorhombic perovskite structure (sp. group *Pbnm*) was observed. A peculiarity of the PrCo_1 − *x*_Fe_*x*_O_3_ solid solution is the lattice parameter crossovers, which occurred at certain compositions and revealed in the pseudo-tetragonal or pseudo-cubic metric. An average crystallite size of the PrCo_1 − *x*_Fe_*x*_O_3_ samples estimated from the analysis of the angular dependence of the X-ray diffraction (XRD) line broadening varies between 30 and 155 nm, depending on the composition and synthesis temperature.

## Background

Complex oxides with perovskite structure *RM*O_3_, where *R* and *M* are rare earth and transition metals, respectively, represent an important class of the functional materials. In particular, the “pure” and mixed rare earth cobaltites and ferrites are used in thermoelectric devices; solid oxide fuel cells, as membranes for partial oxidation of methane; and cleaning oxygen, as catalysts and sensory materials [1–5]. The interest in the rare earth cobaltites *R*CoO_3_ is also stimulated by their unique fundamental physical properties, such as different types of magnetic ordering and temperature-induced metal-insulator (MI) transitions conjugated with the spin-state transitions of Co^3+^ ions [6, 7]. These transitions are strongly affected by the chemical pressure caused by the exchange of cations either in *A*- or *B*-sites of perovskite structure [8–10].

Among the mixed rare earth cobaltites-ferrites *R*Co_1 − *x*_Fe_*x*_O_3_, the most extensively studied is a system with La [10–12], whereas information about phase and structural behaviour in the systems with other rare earths is rather limited. Our recent investigations of structural and thermal behaviour of the mixed cobaltites-ferrites with *R*=Pr, Nd, Sm and Eu obtained by a standard ceramic technique at 1200–1300 °C [13–16] proved a formation of the continuous solid solution with the orthorhombic perovskite structure. In situ high-temperature X-ray synchrotron powder diffraction revealed strong anomalies in the lattice expansion, which are especially pronounced in cobalt-rich specimens. They are reflected in a sigmoidal dependence of the unit cell dimensions, in extra increment of the unit cell volume and in clear maxima of the thermal expansion coefficients [16–19]. These anomalies are related to the changes in spin state of Co^3+^ ions and conjugated MI transitions. They become less pronounced with the decreasing of the cobalt content in the *R*Co_1 − *x*_Fe_*x*_O_3_ series.

Here, we report the results of structural characterization of nanocrystalline cobaltites-ferrites PrCo_1 − *x*_Fe_*x*_O_3_ prepared by sol-gel citrate route.

## Methods

Nanocrystalline powders of PrCo_1 − *x*_Fe_*x*_O_3_ (*x* = 0.1, 0.3, 0.5, 0.7 and 0.9) were prepared by sol-gel citrate method. Crystalline Pr(NO_3_)_3_·6H_2_O (99.99 %, Alfa Aesar), Co(NO_3_)_2_·6H_2_O (ACS, Alfa Aesar), Fe(NO_3_)_3_·9H_2_O (ACS, Alfa Aesar) and a citric acid (CC) were dissolved in water and mixed in the molar ratio of *n*(Pr^3+^):*n*(Co^2+^):*n*(Fe^3+^):*n*(CC) = 1:(1 − *х*):*х*:4 according to the PrCo_1 − *x*_Fe_*x*_O_3_ nominal compositions. Prepared solutions were gelled at ~90 °C and subsequently treated at the temperatures of 700 and 800 °C for 2 h. Thus, two series of the samples were obtained. Spot-check examination of the cationic composition of the samples was performed by energy dispersive X-ray fluorescence (EDXRF) analysis by using XRF Analyzer Expert 3L.

Laboratory X-ray powder diffraction investigation was performed on the Huber imaging plate Guinier camera G670 (Cu *K*_α1_ radiation, *λ* = 1.54056 Å). The high-resolution X-ray synchrotron powder diffraction examination was performed for the PrCo_0.5_Fe_0.5_O_3_@700 °C and PrCo_0.5_Fe_0.5_O_3_@800°C samples with equiatomic amount of Co and Fe. Corresponding experiments were carried out at the beamline ID22 of ESRF (Grenoble, France) during the beamtime allocated to the Experiment N° MA-2320. All crystallographic calculations were performed by means of the programme package WinCSD [20], which was also used for the evaluation of microstructural parameters of the samples. The average grain size of the powders (*D*) and lattice strains <*ε*> = <*Δd*>/*d* were estimated from the analysis of angular dependence of the X-ray diffraction (XRD) profile broadening by using the external Si standard for the correction of instrumental broadening. The morphology of the nanoaggregates was investigated by scanning electron microscopy (SEM) by means of an ESEM FEI Quanta 200 FEGi system operated in a low-vacuum mode (70 Pa) and at an acceleration voltage of 15 kV (FEI Company, Eindhoven, NL). The samples were mounted onto conductive carbon tapes adhered on aluminium holders. High-resolution images were obtained using an Everhart-Thornley detector (ETD) for secondary electrons or a solid-state backscattered electron (SSD-BSE) detector.

## Results and Discussion

**Fig. 1:**
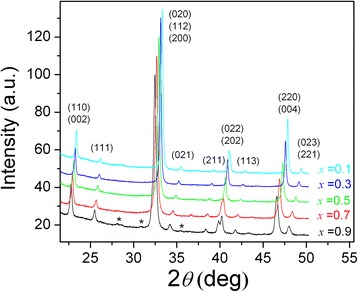
X-ray powder diffraction patterns of PrCo_1 − *x*_Fe_*x*_O_3_ samples synthesized at 800 °C

According to X-ray powder diffraction examination of both series of the mixed cobaltites-ferrites PrCo_1 − *x*_Fe_*x*_O_3_ obtained at 700 and 800 °C, all the samples synthesized were single phase and possess an orthorhombic perovskite structure isotypic with GdFeO_3_ (Fig. 1). Only in the iron-rich specimen PrCo_0.1_Fe_0.9_O_3_@800°C the traces of the unidentified parasitic phases could be detected. EDXRF examination of the sample with nominal composition PrCo_0.5_Fe_0.5_O_3_ revealed 70.96(9) wt.% of Pr, 14.98(7) wt.% of Co and 14.06(7) wt.% of Fe, which corresponds to 0.998(2):0.503(3):0.499(3) molar ratio of the metal components.

Full profile Rietveld refinement, performed in space group *Pbnm*, led to an excellent agreement between calculated and experimental profiles for all PrCo_1 − *x*_Fe_*x*_O_3_ samples. In the refinement procedure, the unit cell dimensions and positional and displacement parameters of atoms were refined together with background and peak profile parameters and correction of absorption and instrumental zero shift. No significant difference in the structural parameters was found between two series of the samples.

Precise high-resolution X-ray synchrotron powder diffraction examination confirms phase purity of PrCo_0.5_Fe_0.5_O_3_ samples obtained at 700 and 800 °C. No traces of foreign phases were detected in both samples even applying this very sensitive diffraction technique.

In spite of superb resolution (typical HWFM of the reflections of Si standard are in the limits of 0.003–0.006 2*θ*^o^), no reflection splitting was observed at the PrCo_0.5_Fe_0.5_O_3_@700°C and PrCo_0.5_Fe_0.5_O_3_@800°C diffraction patterns due to the rather pseudo-cubic metric of the orthorhombic lattice and essential nanocrystalline size effect on the XRD line broadening.

**Fig. 2:**
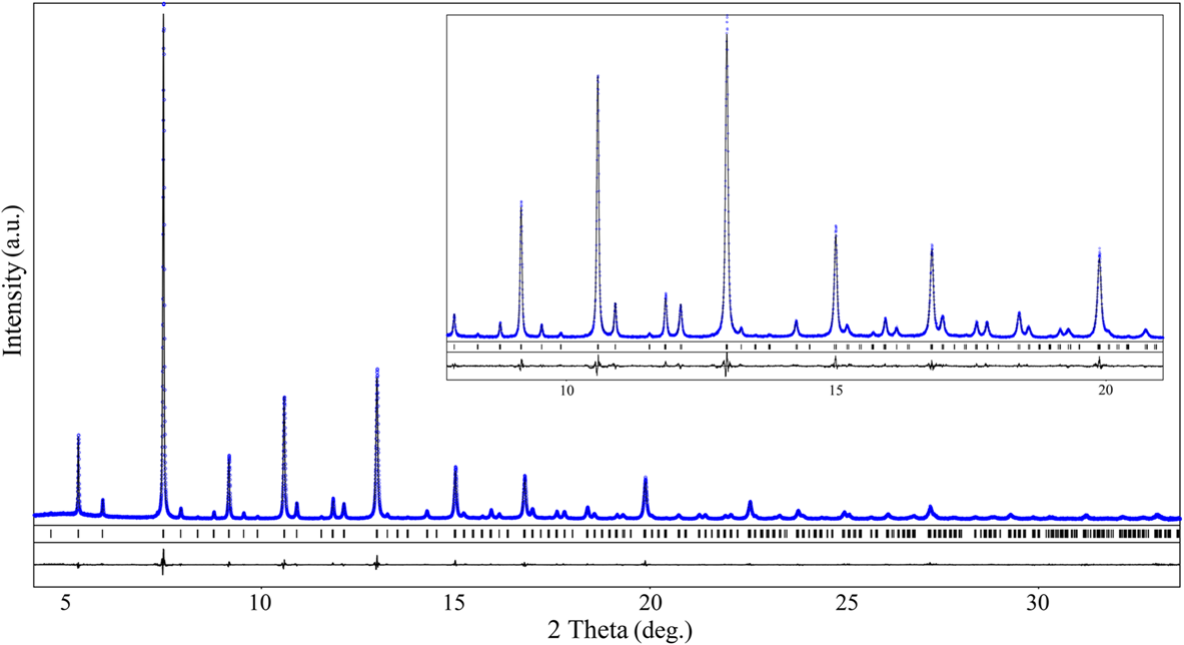
X-ray synchrotron powder diffraction pattern of PrCo_0.5_Fe_0.5_O_3_@800 °C (*λ* = 0.35434 Å). Experimental (*dots*) and calculated patterns; difference profiles and positions of the diffraction maxima are given

However, structural parameters of both samples were successfully refined by the full profile Rietveld method in space group *Pbnm*. As an example, the graphical results of the Rietveld refinement of PrCo_0.5_Fe_0.5_O_3_@800 °C structure are presented in Fig. 2.

Table 1 contains the results of the Rietveld refinement of the PrCo_1 − *x*_Fe_*x*_O_3_ samples obtained at 800 °C by using laboratory X-ray and synchrotron powder diffraction data.

**Table 1:** Lattice parameters, coordinates and displacement parameters of atoms in PrCo_1 − *x*_Fe_*x*_O_3_ (space group *Pbnm*)

Atoms, sites	Parameters, residuals	*x* in PrCo_1 − *x*_Fe_*x*_O_3_					
		0.1	0.3	0.5 (Lab-XRD)	0.5 (Synch-XRD)	0.7	0.9
	*a*, Å	5.3845(2)	5.4044(2)	5.4281(2)	5.4290(1)	5.4544(2)	5.4767(2)
	*b*, Å	5.3559(2)	5.3944(1)	5.4406(2)	5.4413(1)	5.4980(2)	5.5519(2)
	*c*, Å	7.5903(3)	7.6297(2)	7.6735(3)	7.6759(2)	7.7246(3)	7.7699(3)
Pr, 4*c*	*x*	−0.0035(4)	−0.0042(4)	−0.0026(7)	−0.0059(2)	−0.0057(4)	−0.0066(3)
	*y*	0.02944(9)	0.03134(9)	0.0334(1)	0.03285(8)	0.0380(1)	0.0427(1)
	*z*	¼	¼	¼	¼	¼	¼
	*B*_iso_, Å^2^	0.78(1)	1.02(1)	1.02(2)	0.699(6)	1.18(2)	1.19(2)
Fe/Co, 4*b*	*x*	0	0	0	0	0	0
	*y*	½	½	½	½	½	½
	*z*	0	0	0	0	0	0
	*B*_iso_, Å^2^	0.94(2)	1.03(2)	0.64(3)	0.43(2)	0.71(3)	0.61(3)
O1, 4*c*	*x*	0.053(2)	0.036(2)	0.031(3)	0.063(2)	0.044(2)	0.091(2)
	*y*	0.4966(10)	0.4960(8)	0.4992(11)	0.4866(10)	0.4926(10)	0.4856(12)
	*z*	¼	¼	¼	¼	¼	¼
	*B*_iso_, Å^2^	0.37(8)	0.656(3)	0.885(4)	1.8(3)	0.857(2)	0.977(3)
O2, 8*d*	*x*	−0.2937(12)	−0.3070(12)	−0.302(2)	−0.2866(11)	−0.3017(14)	−0.2767(15)
	*y*	0.2772(14)	0.2771(14)	00.277(2)	0.2787(11)	0.2861(14)	0.3013(12)
	*z*	0.0382(10)	0.0440(10)	0.0496(13)	0.0426(8)	0.4482(10)	0.4651(11)
	*B*_iso_, Å^2^	0.37(8)	0.656(2)	0.885(3)	0.75(12)	0.857(2)	0.977(2)
	*R*_*I*_	0.042	0.047	0.054	0.039	0.061	0.043
	*R*_*P*_	0.088	0.092	0.123	0.091	0.112	0.125

**Fig. 3:**
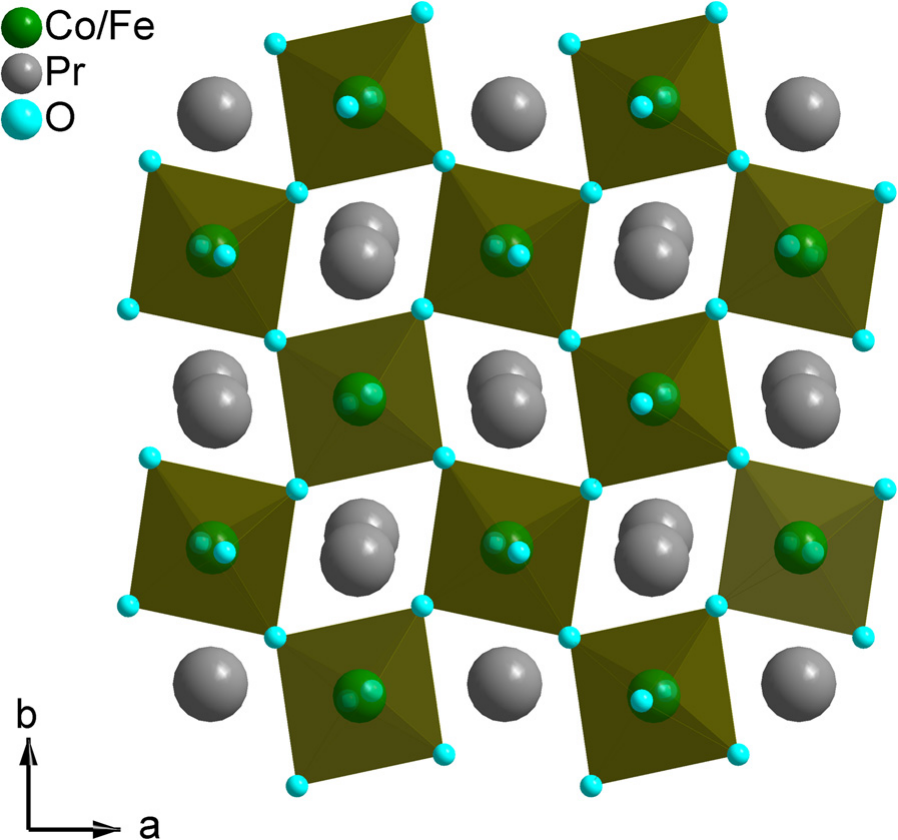
Projection of PrCo_0.5_Fe_0.5_O_3_ structure on (001) plane

Similar to the “pure” PrCoO_3_ and PrFeO_3_ compounds, crystal structure of the mixed cobaltites-ferrites PrCo_1 − *x*_Fe_*x*_O_3_ can be described as a framework of corner-shared *M*O_6_ (*M* = Co/Fe) octahedra with the Pr atoms occupying holes between them. A projection of the PrCo_0.5_Fe_0.5_O_3_ structure along [001]-direction is shown in Fig. 3.

**Fig. 4:**
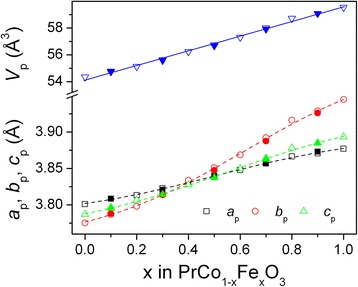
Concentration dependencies of the normalized unit cell dimensions of PrCo_1 − *x*_Fe_*x*_O_3_ series. *Solid* and *open symbols* correspond to the samples synthesized by sol-gel technique at 800 °C and by solid-state reactions at 1300 °C, respectively. The *dashed lines* are guide for the eyes. The lattice parameters of the orthorhombic cell are normalized to the perovskite one as follows: *a*_*p*_ *= a*_*o*_/√2, *b*_*p*_ *= b*_*o*_/√2, *c*_*p*_ *= c*_*o*_/2, *V*_*p*_ *= V*_*o*_/4

The analysis of the concentration dependence of the unit cell dimensions of the sol-gel-obtained PrCo_1 − *x*_Fe_*x*_O_3_ samples (Fig. 4, solid symbols) proves the formation of continuous solid solution, similar to those observed recently for the mixed praseodymium cobaltites-ferrites obtained by standard ceramic technique (Fig. 4, open symbols) [13, 16]. Peculiarity of the PrCo_1 − x_Fe_x_O_3_ solid solution is a lattice parameter crossover that occurred at a certain composition, which becomes apparent in the pseudo-tetragonal or pseudo-cubic unit cell dimensions (Fig. 4). The reason for this phenomenon, which was earlier also observed in the related rare earth aluminates, gallates and titanates-chromites [21–26], is the different cell parameter ratios within the same orthorhombic GdFeO_3_ type of structure observed for the end members of these series.

**Fig. 5:**
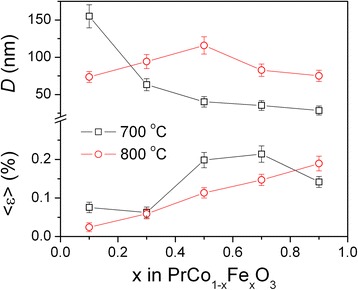
Microstructural parameters of the PrCo_1 − *x*_Fe_*x*_O_3_ series synthesized at 700 and 800 °C

Microstructural parameters of two PrCo_1 − *x*_Fe_*x*_O_3_ series synthesized at 700 and 800 °C were evaluated from the analysis of the XRD profile broadening by using the external Si standard. Average grain size *D* in both series is estimated to vary within the limit of 30–155 nm, depending on the composition and synthesis temperature (Fig. 5). The *D* values in the PrCo_1 − *x*_Fe_*x*_O_3_@700°C samples systematically decrease with the increasing of iron content, whereas in the PrCo_1 − *x*_Fe_*x*_O_3_@800°C series, this parameter goes through the maximum at *x* = 0.5. In both series, the increase of the lattice strains is observed with increasing iron content (Fig. 5).

**Fig. 6:**
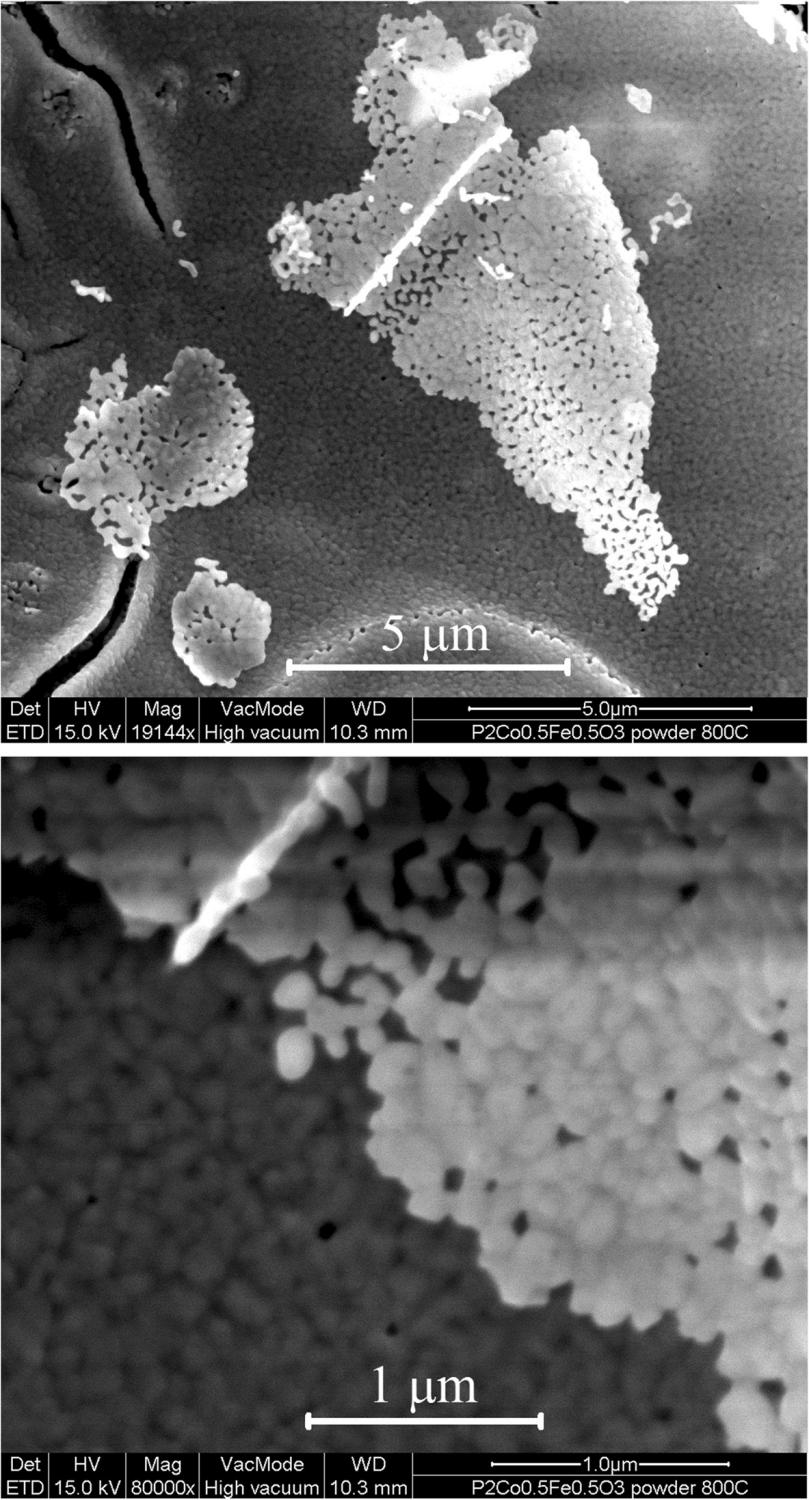
Scanning electron microscopy of PrCo_0.5_Fe_0.5_O_3_@800 °C. Both images were done with secondary electrons

Scanning electron microscopy investigation of the PrCo_0.5_Fe_0.5_O_3_ sample prepared at 800 °C (Fig. 6) revealed a lacy morphology of the powder consisting of irregular shaped 60–100-nm nanoparticles.

## Conclusions

Two series of the nanocrystalline mixed cobaltites-ferrites PrCo_1 − *x*_Fe_*x*_O_3_ (*x* = 0.1, 0.3, 0.5, 0.7 and 0.9) of high phase purity were prepared by sol-gel citrate method at 700 and 800 °C. The average grain size of the powders estimated from the analysis of angular dependence of the XRD line broadening varies between 30 and 155 nm, depending on the composition and synthesis temperature. Refined structural parameters of the PrCo_1 − *x*_Fe_*x*_O_3_@700 °C and PrCo_1 − *x*_Fe_*x*_O_3_@800 °C series prove the formation of continuous solid solution as it was shown earlier for the similar series obtained by the standard ceramic technique at 1300 °C. In comparison with a traditional energy- and time-consuming high-temperature solid-state synthesis technique, the low-temperature sol-gel citrate method is a very promising tool for the obtaining of fine powders of the mixed perovskite oxide materials, free of contamination of constituent metal oxides or other parasitic phases.
